# Association of N^6^-methyladenine DNA with plaque progression in atherosclerosis via myocardial infarction-associated transcripts

**DOI:** 10.1038/s41419-019-2152-6

**Published:** 2019-12-04

**Authors:** Lianpin Wu, Yuqing Pei, Yinhuan Zhu, Minghua Jiang, Cheng Wang, Wei Cui, Donghong Zhang

**Affiliations:** 1 Department of Cardiology, The Second Affiliated Hospital of Wenzhou Medical University, 109 Xueyuan Road, Wenzhou, 325027 Zhejiang China; 2 Clinical Laboratory, National Cancer Center/National Clinical Research Center for Cancer/Cancer Hospital, Chinese Academy of Medical Sciences and Peking Union Medical College, Beijing, 100021 China; 3 Clinical Laboratory, The Second Affiliated Hospital of Wenzhou Medical University, 109 Xueyuan Road, Wenzhou, 325027 China; 4 Clinic Center of Human Gene Research, Union Hospital of Tongji Medical College, Huazhong University of Science and Technology, Wuhan, 430000 China; 5 Center for Molecular and Translational Medicine, GeorgiaELISA analysis of global m6A level with ALKBH1 State University, Research Science Center, 157 Decatur St SE, Atlanta, GA 30303 USA

**Keywords:** Diagnostic markers, Atherosclerosis

## Abstract

Modification of the novel N^6^-methyladenine (m6A) DNA implicates this epigenetic mark in human malignant disease, but its role in atherosclerosis (AS) is largely unknown. Here, we found that the leukocyte level of m6A but not 5mC DNA modification was decreased with increasing of carotid plaque size and thickness in 207 AS patients as compared with 142 sex- and age-matched controls. Serum low-density lipoprotein (LDL) and leukocyte m6A levels were associated with the progression of carotid plaque size and thickness. Both LDL level and plaque thickness were also independently and negatively related to m6A level. Reduced m6A level was further confirmed in leukocytes and endothelium in western diet-induced AS mice and in oxidized-LDL (ox-LDL)-treated human endothelium and monocyte cells. Decreased m6A level was closely related to the upregulation of AlkB homolog 1 (ALKBH1), the demethylase of m6A. Silencing of ALKBH1 or hypoxia-inducible factor 1α (HIF1α) could rescue the ox-LDL–increased level of MIAT, a hypoxia-response gene. Mechanically, ox-LDL induced HIF1α for transfer into the nucleus. Nuclear HIF1α bound to the ALKBH1-demethylated MIAT promoter and transcriptionally upregulated its expression. Therefore, elevated ALKBH1 level in endothelium and leukocytes reduced m6A level, which is a novel and sensitive biomarker for AS progression.

## Introduction

Atherosclerosis (AS) is the major cause of cardiovascular disease (CVD), the leading cause of morbidity and mortality globally^1,2^. Most events derive from the rupture or erosion of atherosclerotic plaque. The progression of carotid intima media thickness (CIMT) in the common carotid artery or the presence of carotid plaques could be surrogate markers of AS^3^. CIMT can be evaluated non-invasively by using carotid B-mode ultrasonography, considered the most reliable and reproducible method to visualize all CIMT segments^4,5^.

Emerging evidence has suggested that AS is also an epigenetic disease with the interplay of multiple epigenetic mechanisms, such as DNA methylation, histone acetylation, microRNAs and long non-coding RNAs (lncRNAs)^6^. Exposure to a plethora of environmental pollutants induced epigenetic modifications of gene expression relevant to the onset or progression of CVD^7,8^. 5-methylcytosine (5mC) DNA methylation is a major epigenetic mechanism. We have much evidence for altered global and locus-specific 5mC patterns in human atherosclerotic lesions or leukocytes^2,9,10^ but with contrasting results.

In addition to 5mC DNA methylation, DNA adenine methylation (N^6^-methyl-2’-deoxyadenosine [m6A]) is a naturally occurring DNA modification preserved in prokaryotes to eukaryotes^11^. m6A DNA modification in the human genome is mediated by the methyltransferase N-6 adenine-specific DNA methyltransferase 1 (N6AMT1) and demethylase AlkB homolog 1 (ALKBH1)^12^. Current evidence shows that dynamic m6A modification in genomic DNA is associated with brain functions^13^, glioblastoma^14^, embryogenesis^15^, reproduction^16^, and embryonic stem cell development^17^. However, whether m6A modification plays a role in gene regulation and disease pathogenesis, including CVD, remains largely unexplored.

Recent study showed that hypoxia-induced genes were modulated by ALKBH1-related m6A in human glioblastoma^14^. The lncRNA myocardial infarction-associated transcript (MIAT), as a hypoxia-response gene, was reported as a target gene of ALKBH1-modulated m6A in glioblastoma. MIAT has an oncogenic role^18–20^, and elevated MIAT level aggravated atherosclerotic damage in mice with AS^21^ and angiotensin II-induced cardiac hypertrophy^22^. Although hypoxia-response genes such as MIAT might be the target genes of ALKBH1-related m6A modification in glioblastoma, the profiling of m6A and the underlying mechanism of MIAT regulation by m6A during AS development is unknown.

In this study, we sought to determine the profile of global 5mC and m6A DNA methylation and their role in different stages of AS. We found a marked decrease in leukocyte and endothelial m6A level but not 5mC DNA methylation closely related to human and mouse plaque progression. Oxidized low-density lipoprotein (ox-LDL)-induced ALKBH1 and m6A DNA demethylation further facilitated hypoxia-inducible factor 1α (HIF1α) binding and activation of MIAT. Thus, m6A DNA methylation could be a sensitive marker predicting the progression of plaque via MIAT regulation.

## Results

### Reduced leukocyte m6A DNA level is associated with plaque progression in AS patients

**Fig. 1:**
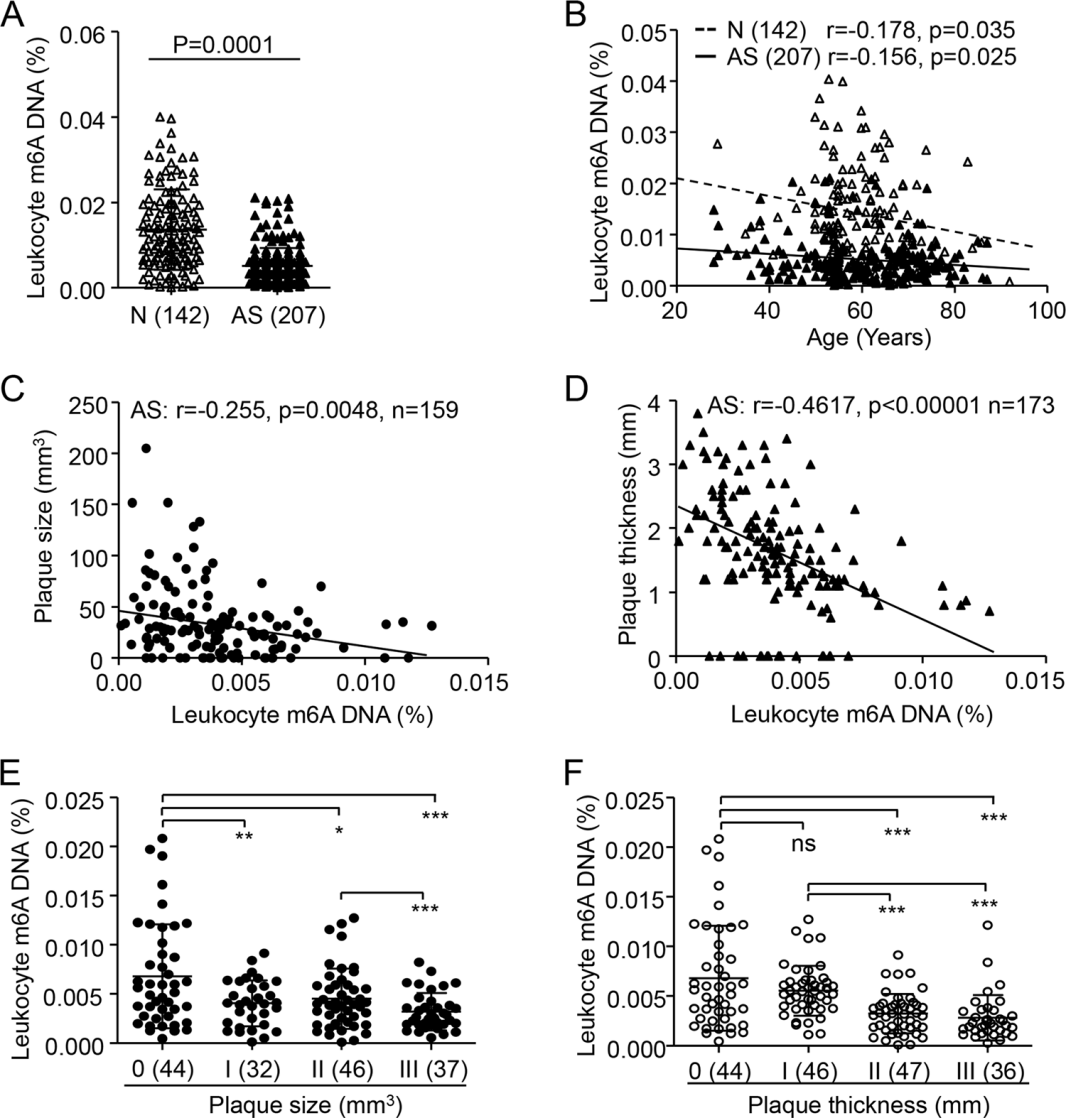
Decreased leukocyte N6-methyladenosine (m6A) DNA level is associated with carotid plaque progression in patients with atherosclerosis (AS). **a** Leukocyte m6A level in AS patients and normal individuals (N). Data are mean ± SD and were compared by unpaired *t* test. **b**–**d** Spearman correlation coefficients for leukocyte m6A level correlated with age (**b**), carotid plaque size (**c**), and carotid intima media thickness (CIMT) (**d**). Overall carotid plaque size (**e**) and CIMT (**f**) by leukocyte m6A level. Data are mean ± SD and were compared by one-way ANOVA, followed by Bonferroni’s multiple comparison test. *P < 0.05, **P < 0.01, ***P < 0.001. ns not significant.

We first determined global m6A and 5mC DNA methylation in peripheral blood from 207 patients with clinical AS and 142 age- and sex-matched controls. Leukocyte m6A level was significantly lower in AS patients than controls by about 67.3% (mean 0.04 ± 0.002% vs. 0.013 ± 0.004%) (Fig. 1a). Reduced m6A level was related to age in both controls and AS patients (Fig. 1b) but not sex in controls or AS patients (data not shown). Leukocyte m6A level was inversely correlated with both carotid plaque size (*r* = −0.255, *p* = 0.0048) and thickness (*r* = −0.4617, *p* < 0.0001) (Fig. 1c, d). Moreover, m6A level was significantly lower in AS patients with than without carotid plaque (Fig. 1e). Patients with the largest or thickest carotid plaque had the lowest m6A level (Fig. 1e, f). However, leukocyte 5mC level was slightly but not significantly lower in AS patients than controls (Fig. S1A). 5mC level was gradually reduced with age only in AS patients (Fig. S1B). Furthermore, leukocyte 5mC level was not correlated with carotid plaque size or thickness in AS patients (Fig. S1C, D). Therefore, decreased leukocyte m6A but not 5mC level was associated with plaque progression in AS patients.

### Serum LDL level is negatively associated with leukocyte m6A level during plaque progression

We next detected the association between reduced leukocyte m6A DNA level and biochemical characteristics of AS patients. Linear regression analysis revealed leukocyte m6A level negatively associated with plaque size, plaque thickness, and serum homocysteine (Hcy) and LDL levels but positively with vitamin B12 (VB12) and albumin levels after adjustment for age and sex (Table 1). Multivariable regression analysis further confirmed that leukocyte m6A level was inversely related to LDL levels and plaque thickness but positively with VB12 level (Table 2) when including all variables by stepwise multivariable analysis. Conversely, logistic regression analysis revealed an association between increased Hcy, LDL, and apolipoprotein B (ApoB) levels and decreased m6A level with plaque size and CIMT progression (Table 3). However, 5mC level was not related to plaque progression in AS patients (Tables 1 and 2). Therefore, serum LDL level was negatively associated with leukocyte m6A level during the progression of cortical plaque size and CIMT in AS patients.

**Table 1:** Linear regression analysis of association of clinical factors and DNA methylation adjusted for age and sex.

Clinical factors	m6A DNA			5mC DNA		
	*β*	95% CI	*P*	*β*	95% CI	*P*
Plaque size (mm^3^)	−0.289	−0.825 to −0.068	**<0.001**	−0.076	−0.347 to 0.127	0.337
CIMT (mm)	−0.443	−1.124 to −0.103	**<0.001**	−0.065	−0.170 to 0.115	0.396
Hcy (µmol/L)	−0.162	−0.423 to −0.024	**0.023**	−0.115	−0.521 to −0.025	**0.031**
VB12 (pg/ml)	0.171	0.012 to 0.424	**0.017**	−0.025	−0.103 to 0.087	0.730
Folic acid (ng/ml)	0.082	−0.143 to 0.247	0.259	0.031	−0.129 to 0.294	0.662
Albumin (g/L)	0.257	0.019 to 0.532	**<0.001**	−0.008	−0.303 to 0.387	0.911
LDL (mmol/L)	−0.175	−0.494 to −0.089	**0.023**	0.020	−0.126 to 0.272	0.776
TG (mmol/L)	0.057	−0.197 to 0.202	0.426	−0.089	−0.301 to 0.251	0.208
HDL (mmol/L)	0.084	−0.102 to 0.347	0.235	−0.026	−0.378 to 0.324	0.706
ALT (U/L)	−0.077	−0.236 to 0.173	0.282	0.044	−0.208 to 0.325	0.530
AST (U/L)	−0.062	−0.279 to 0.368	0.383	0.019	−0.107 to 0.305	0.788
ALP (U/L)	−0.035	−0.207 to 0.159	0.616	0.040	−0.246 to 0.301	0.561
Urea (mmol/L)	−0.057	−0.349 to 0.267	0.420	−0.061	−0.142 to 0.108	0.378
UA (µmol/L	0.115	−0.134 to 0.360	0.103	0.107	−0.214 to 0.480	0.125
Apo A (g/L)	0.075	−0.141 to 0.342	0.328	−0.040	−0.237 to 0.253	0.592
Apo B (g/L)	−0.072	−0.416 to 0.504	0.332	0.035	−0.241 to 0.358	0.635
TC (mmol/L)	0.051	−0.312 to 0.465	0.487	−0.042	−0.475 to 0.349	0.557

*CIMT* carotid intima-media thickness, *Hcy* homocysteine, *VB12* vitamin B12, *LDL* low-density lipoprotein, *TG* triglycerides, *HDL* high-density lipoprotein, *ALT* alanine aminotransferase, *AST* aminotransferase, *ALP* alkaline phosphatase, *Apo A* apolipoprotein A, *Apo B* apolipoprotein B, *TC* total cholesterol

The bold, significant difference

**Table 2:** Multivariate model for the association of selected clinical features and DNA methylation.

Clinical factors	m6A DNA			5mC DNA		
	*β*	95% CI	*P*	*β*	95% CI	*P*
CIMT	−0.391	−0.679 to −0.103	**<0.001**	−0.087	−0.211 to 0.274	0.270
LDL	−0.898	−0.298 to −8.011	**0.046**	0.376	0.781 to 0.093	**0.025**
VB12	0.170	0.084 to 0.349	**0.025**	0.069	−0.143 to 0.293	0.381
Hcy	−0.351	−0.812 to 0.059	0.310	−0.228	−0.561 to −0.007	**0.034**

*CIMT* carotid intima-media thickness, *VB12* vitamin B12, *LDL* low-density lipoprotein, *Hcy* homocysteine

**Table 3:** Logistic regression analysis of the association of clinical factors and plaque.

Clinical factors	Plaque size			CIMT		
	OR	95% CI	*P*	OR	95% CI	*P*
Hcy	0.699	0.110 to 2.801	**<0.001**	0.742	0.149 to 3.848	**<0.001**
m6A DNA	−1.605	−2.345 to −0.098	**0.015**	−1.498	−2.129 to −0.054	**0.024**
LDL	0.991	0.084 to 5.998	**0.011**	0.990	0.013 to 3.997	**0.005**
Apo B	18.435	2.277 to 149.228	**0.006**	12.119	1.607 to 91.387	**0.016**

*Hcy* homocysteine, *LDL* low-density lipoprotein, *Apo B* apolipoprotein B

### Elevated ALKBH1 level in leukocytes and endothelium reduced m6A DNA level in vivo and in vitro

**Fig. 2:**
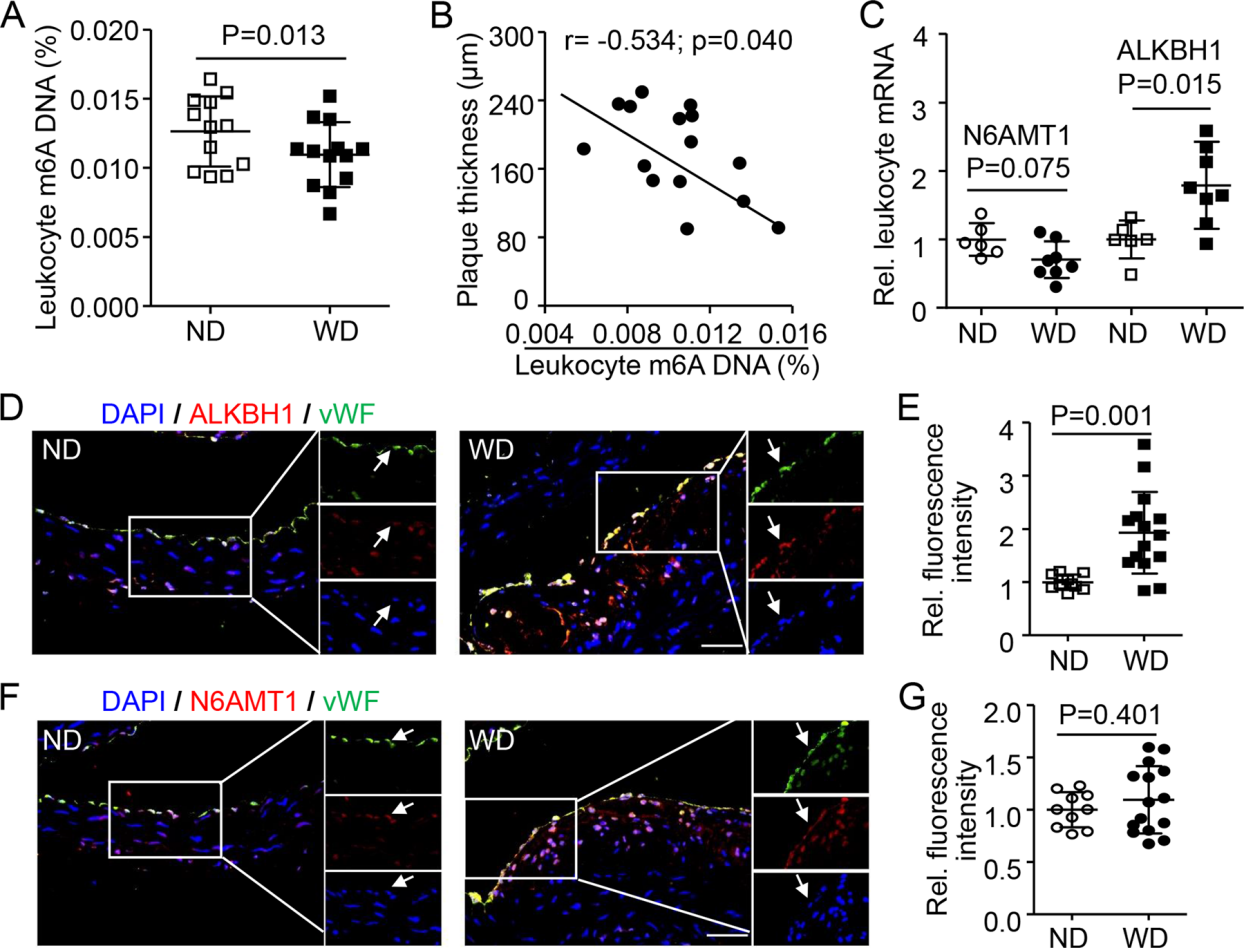
Effect of m6A DNA and its modulators in leukocytes and vasculature of mice with atherosclerosis (AS) induced by a western diet (WD). **a** Overall leukocyte m6A levels in male ApoE^−/−^ mice (8 weeks old) fed a WD (*n* = 13) and normal diet (ND, *n* = 12) for 6 months. **b** Spearman correlation coefficient for leukocyte m6A level correlated with plaque thickness of aortic root (*n* = 15). **c** mRNA expression of N6AMT and ALKBH1 in leukocytes (*n* = 6 for ND; *n* = 8 for WD). **d**–**g** Representation immunofluorescence co-staining and quantification of ALKBH1 (**d**, **e**) and N6AMT1 (**f**, **g**) in endothelial cells (ECs) of frontal sections from aortic root (*n* = 10 for ND; *n* = 15 for WD). Scale bar: 50 µm. von Willebrand factor (vWF) was the EC marker. Data are mean ± SD and were compared by unpaired *t* test in (**a**, **c**, **b**, and **g**). Rel. Relative, DAPI was a nuclear marker.

To explore the response of m6A during plaque progression in vivo, we generated a mouse aortic root AS model induced by a western diet (WD) for 6 months in apolipoprotein E (ApoE)-knockout mice. Consistent with the observations in AS individuals, leukocyte m6A DNA level was lower in WD-induced AS mice than mice fed a normal diet (ND) (Fig. 2a). The reduced leukocyte m6A level was inversely correlated with aortic root plaque thickness in AS mice (*r* = −0.534; *p* = 0.040) (Fig. 2b). In addition, as compared with ND mice, AS mice showed significantly increased leukocyte mRNA level of ALKBH1, the demethyltransferase of m6A, and slightly decreased level of N6AMT1, the methyltransferase of m6A (Fig. 2c). Especially, on immunofluorescence co-staining, ALKBH1 protein level was greatly decreased in endothelium, but not vascular smooth muscle cells or microphages of aortic root plaque of AS vs. ND mice (Figs. 2d, e and S2A, B). We found no significant changes in N6AMT1 level in WD-treated aortic root plaque (Figs. 2f, g and S2C, D). Similarly, global leukocyte 5mC DNA methylation was not associated with WD-induced plaque thickness (Fig. S3A, B). As well, AS mice and ND mice did not differ in 5mC level in endothelium, vascular smooth muscle cells or microphages of aortic root plaque (Fig. S3C–E).

**Fig. 3:**
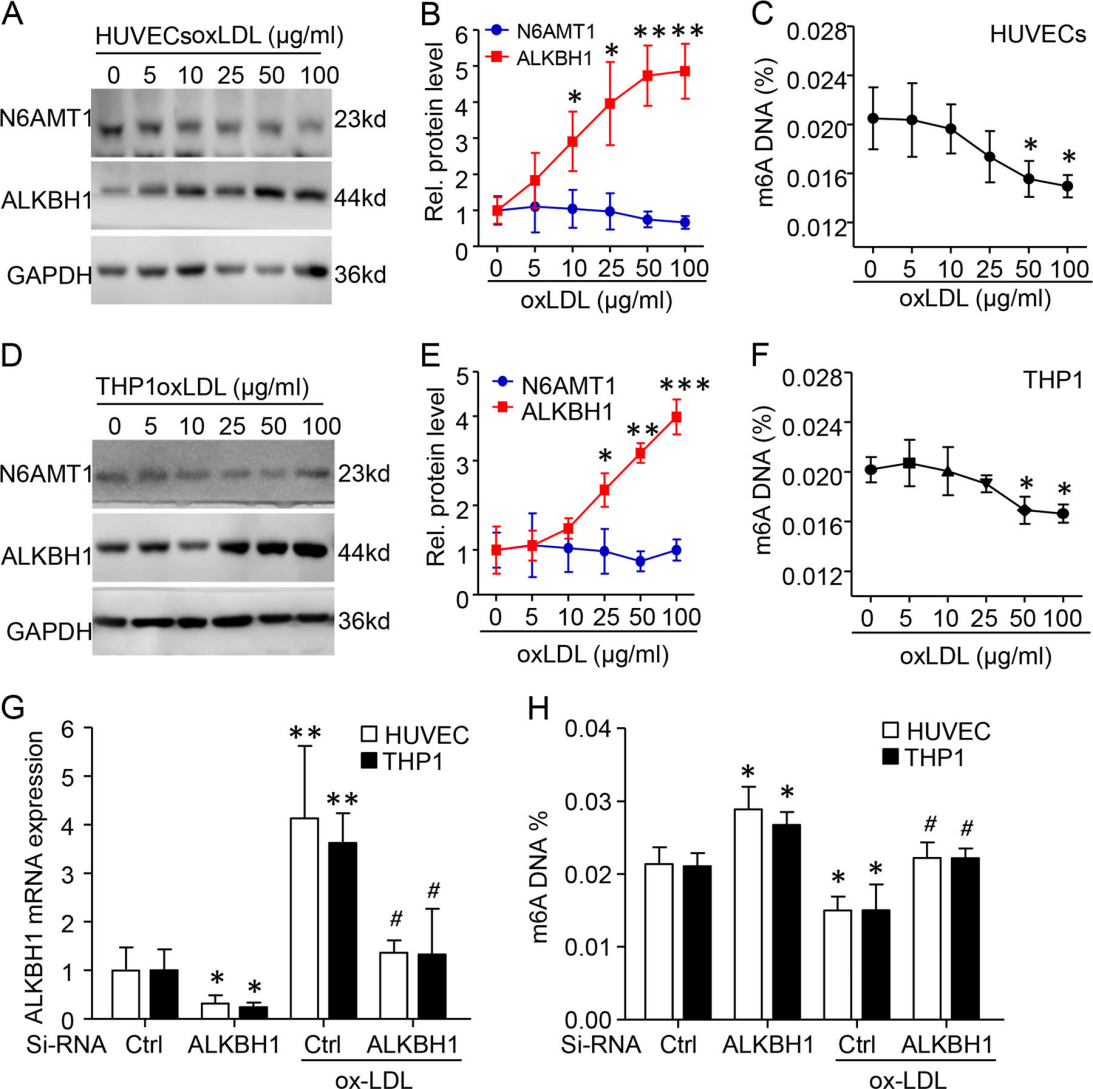
Elevated ALKBH1 level in leukocytes and endothelium reduced m6A DNA in vitro. Human umbilical vein endothelial cells (HUVECs) and human monocytic cells (THP-1) were treated with various concentrations of oxidized low-density lipoprotein (ox-LDL) for 24 h. **a**–**f** Representative immunoblotting and quantification of N6AMT1 and ALKBH1 levels and global m6A level in ox-LDL–treated HUVECs (**a**–**c**) and THP-1 cells (**d**–**f**). GAPDH was the internal control. **g** qRT-PCR assay of ALKBH1 mRNA level with siRNA-Ctrl (Control) or siRNA-ALKBH1 transfection with or without ox-LDL (50 μg/ml) treatment in HUVECs and THP-1 cells. **h** ELISA analysis of global m6A level with ALKBH1 knock down in ox-LDL-treated HUVECs and THP-1 cells. Data are mean ± SD (*n* = 4/group) and were analyzed by one-way ANOVA, followed by Bonferroni’s multiple comparison test. *P < 0.05, **P < 0.01. ***P < 0.001 vs. 0 µg/ml ox-LDL. ^#^*P* < 0.05 vs. 50 µg/ml ox-LDL for **g** and **h**.

We next determined the regulation of m6A DNA level in vitro. HUVECs and THP1 cells were simulated with ox-LDL, an independent risk factor for m6A and plaque (Tables 2 and 3). Western blot and ELISA assay revealed a dose-dependent upregulation of ALKBH1 but not N6AMT1 with ox-LDL treatment in both cells; global m6A levels were reduced at the high concentration of ox-LDL, 50–100 µmol/L (Fig. 3a–f). Importantly, silencing of ALKBH1 by siRNA transfection could recover ox-LDL-reduced m6A DNA level in both of HUVECs and THP1 cells (Fig. 3g, h). Thus, elevated ALKBH1 level in leukocytes and endothelium directly decreased the m6A DNA level.

### lncRNA MIAT was the target gene of m6A DNA modification in the AS mouse model

**Fig. 4:**
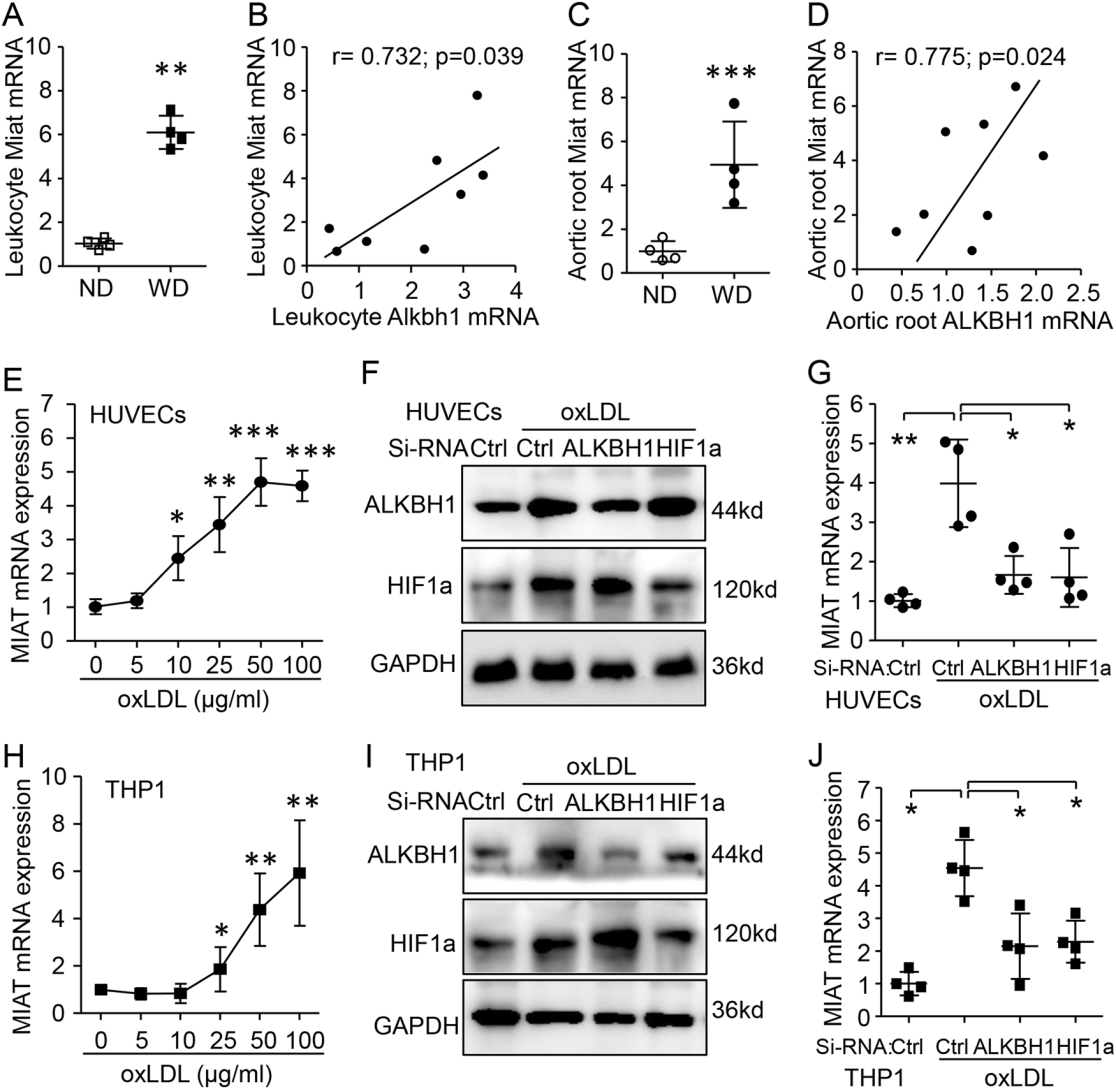
Regulation of myocardial infarction-associated transcript (MIAT) by ALKBH1 and HIF1α under ox-LDL treatment. **a**–**d** *Miat* mRNA relative expression and correlation with ALKBH1 level in leukocytes and aortic root tissue in WD-induced AS mice (*n* = 4/group). **e** qRT-PCR assay of *MIAT* mRNA level with ox-LDL treatment in HUVECs. **f**, **g** Western blot assay of ox-LDL-induced ALKBH1 or HIF1α protein levels (**f**) and qRT-PCR assay of *MIAT* mRNA level (**g**) with siRNA targeting ALKBH1 or HIF1a pre-transfection in HUVECs. **h** qRT-PCR assay of *MIAT* mRNA level with ox-LDL treatment in THP1 cells. **i**, **j** Western blot assay of ox-LDL-induced ALKBH1 or HIF1α protein level (**i**) and qRT-PCR assay of *MIAT* mRNA level (**j**) with siRNA targeting ALKBH1 or HIF1a pre-transfection in THP1 cells. Data are mean ± SD (*n* = 4/group) and were analyzed by one-way ANOVA, followed by Bonferroni’s multiple comparison test. *P < 0.05, **P < 0.01. ***P < 0.001 vs. ND (**a**, **c**) or without ox-LDL treatment in (**e**, **g**, **h** and **j**).

Previous study revealed ALKBH1-sensitive, m6A-site, signature hypoxia-response genes, including lncRNA MIAT, in human glioblastoma^14^. Consistent with previous studies^21,23,24^, we also found elevated *Miat* level in leukocytes and aortic root tissue of the AS mouse model. In addition, *Miat* mRNA expression was positively associated with ALKBH1 level in both cell types (Fig. 4a–d). Furthermore, ox-LDL dose-dependently increased the expression of MIAT in HUVECs and THP1 cells (Fig. 4e, h). To study the effect of HIF1α in ox-LDL-induced MIAT expression, we silenced ALKBH1 or HIF1α by siRNA transfection in both cell types (Fig. 4f, i). Ox-LDL-induced MIAT expression could be successfully inhibited by ALKBH1 or HIF1α siRNA knockdown (Fig. 4g, j). Thus, ox-LDL-induced m6A and HIF1α contributed to MIAT upregulation, a risk factor of plaque in AS pathophysiology.

### Ox-LDL-induced m6A demethylation facilitated HIF1α binding and activation of MIAT

**Fig. 5:**
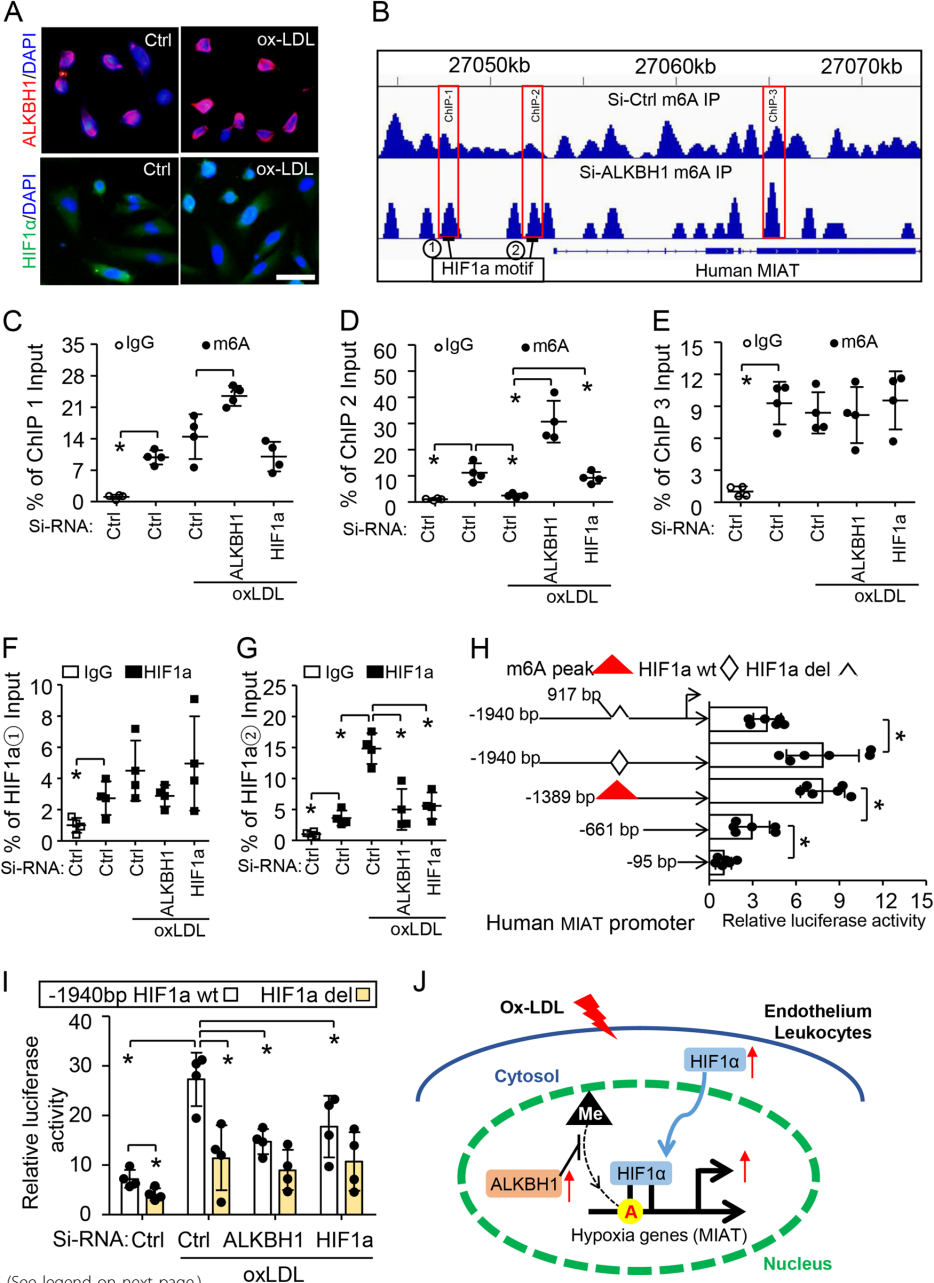
Ox-LDL-induced m6A demethylation promotes HIF1α binding to the MIAT promoter and enhances its activity. **a** Immunofluorescence staining of subcellular localization of ALKBH1 and HIF1α in HUVECs after treatment with 50 μg/ml ox-LDL. Scale bar: 50 µm. DAPI was a nuclear marker. **b** Integrative genomics viewer plots showing the increasing m6A peaks (labeled ChIP1–3) in human *MIAT* gene (hg19) region with ALKBH1 knockdown by siRNA. Two HIF1α motifs on MIAT promoter are numbered. **c**–**h** Chromatin immunoprecipitation (ChIP) assay with m6A (**c**–**e**) or HIF1α (**f**, **g**) antibody used for immunoprecipitation on MIAT fragments in treated HUVECs; normal IgG was an IP control (*n* = 4 per group). **h** Serial deletion constructs of MIAT-Luc with or without HIF1α deletion and pRL vectors were co-transfected into HUVECs. Relative promoter activities were measured by dual-luciferase reporter assay, normalized to Renilla activity (*n* = 5 per group). **i** Elevated luciferase activity in ox-LDL-treated HUVECs prevented by pre-treatment with Si-RNA-ALKBH1 or HIF1α for MIAT plasmids with HIF1α motif (*n* = 4 per group). Data are mean ± SD and were analyzed by one-way ANOVA, Tukey’s test for (**c**–**i**). *P < 0.05, **P < 0.01. **j** Schematic model showing the activation of hypoxia genes by ox-LDL-related m6A signaling in endothelium and leukocytes during atherosclerosis development.

We next revealed the underlying mechanism of ox-LDL regulation of MIAT by cross-linking m6A and HIF1α in HUVECs. Immunofluorescence staining showed that ox-LDL increased nucleic ALKBH1 level and also induced HIF1α for transfer into the nucleus (Fig. 5a). Bioinformatics analysis revealed three m6A peaks around human MIAT gene that were increased by ALKBH1 siRNA knockdown and two HIF1α motifs located in the first and second m6A peaks (Fig. 5b). ChIP assay with m6A antibody confirmed that m6A could bind the three regions (Fig. 5c–e). Only the second m6A peaks were sensitive to ox-LDL treatment because ox-LDL could decrease m6A occupancy on this peak. This m6A peak responded to ALKBH1 and HIF1α silencing, thus preventing ox-LDL-induced m6A binding on this region. ChIP assay with HIF1α antibody further confirmed that ox-LDL-reduced m6A binding could promote HIF1α accumulation on the second but not first m6A peak (Fig. 5f, g).

We then investigated the effect of m6A and HIF1α on MIAT transcriptional activation with ox-LDL treatment. We constructed serial plasmids with or without the second m6A peak for dual-luciferase reporter assay in HUVECs. MIAT transcriptional activation was increased 2.84-fold with than without the m6A peak (Fig. 5h). Deletion of the HIF1α motif in the m6A peak region could fully repress MIAT activation. In addition, ox-LDL treatment could increase MIAT activity with the HIF1α motif (−1940/+166-Luc plasmids) but not with deletion. This upregulation could be largely blocked by siRNA ALKBH1 or HIF1α transfection (Fig. 5i). Therefore, ox-LDL-induced m6A demethylation by ALKBH1 could facilitate HIF1α binding and promote MIAT transcriptional activation (Fig. 5j).

## Discussion

In the current study, we found m6A DNA present in the leukocyte DNA of normal humans. Notably, leukocyte m6A but not 5mC level was significantly decreased in AS patients and gradually reduced with plaque progression. Serum LDL level contributed to dynamic loss of m6A for AS patients. Both m6A and LDL levels also inversely predicted plaque development. Elevated ALKBH1 level, the demethyltransferase of m6A, was essential for reduced m6A level in leukocytes and endothelium in a WD-induced AS mouse model. Mechanistically, ox-LDL-induced m6A DNA demethylation by ALKBH1 could facilitate HIF1α binding and promote MIAT transcription. Thus, our study suggests a potential role for the ALKBH1-m6A regulatory axis in controlling plaque progression via MIAT.

Emerging evidence in the past 2 decades has suggested the importance of epigenetic mechanisms as a new layer of biological regulation in CVD. Genome-wide 5mC DNA methylation studies have identified thousands of differentially methylated sites in human atherosclerotic lesions, but the results remain conflicting. In addition, the most differentially methylated sites show little overlap^6,9^. Similarly, confusing results of 5mC DNA methylation changes were found in peripheral blood cells from human AS patients^25,26^. Consistent with the above observations, our study showed a slight but not significant decrease in 5mC DNA methylation in human AS plaque and leukocytes.

In addition to the canonical 5mC, m6A DNA modification has been newly identified in mammalian genomes. We first found m6A extensively present in human and mouse leukocytes, although relatively rarer than 5mC. In our clinical investigation, leukocyte m6A level was more sensitive than 5mC level to plaque progression. LDL was the independent risk factor for the change in m6A level and plaque progression. Moreover, m6A level in the pathophysiology of WD-induced AS in mice indicated a similar pattern with human endothelial and leukocyte cells. Our evidence further suggested that epigenetics is a systematic change although we did not clearly identify the risk factors of AS, such as lifestyle, diet, or pharmaceutical medications, that contribute to the changes in m6A DNA modification for global epigenetic reprogramming. Thus, m6A rather than 5mC may function as a sensitive potential epigenetic DNA mark in eukaryotes.

The distribution of m6A in the eukaryote genome was reported to be species- and tissue-specific. The human and mouse leukocyte m6A DNA level in our report was comparable to that in primary gastric and liver cancer tissue^14^. Functionally, the ALKBH1-reduced m6A level regulating a hypoxia-response gene signature is associated with tumor grade and reduced patient survival and functions as a therapeutically targetable node^12,14^. The lncRNA MIAT, as a hypoxia-response gene, was reported as the novel target gene of ALKBH1-modulated m6A. Our mechanistic studies revealed that ox-LDL induced m6A demethylation by ALKBH1 on an HIF1α binding region, which facilitated HIF1α binding and promoted MIAT transcriptional activation. Although the HIF1α motif has adenosine and is required for HIF1α binding, we could not precisely identify whether it is modified at N^6^ by methylation. Recent sequencing technology could not distinguish genomic m6A and A. Nevertheless, our current evidence suggests that the function of m6A seems similar to 5mC modification. m6A could prevent some transcriptional factors binding to the gene promoter and suppress gene transcriptional activity. Moreover, this result combined with recent studies further demonstrated that the content and distribution of m6A in human genomic DNA are involved in functional regulation in carcinogenesis and CVD^27–29^, the top two leading causes of morbidity and mortality globally.

In summary, our clinical investigation and experimental study indicated an association of gradually decreased leukocyte genomic m6A but not 5mC DNA modification with plaque progression and the development of AS. LDL was an independent risk factor of decreased m6A level and plaque progression. Furthermore, ox-LDL-induced m6A demethylation by ALKBH1 could facilitate HIF1α binding and promotion of MIAT transcriptional activation. m6A, a new class of DNA modification, may have a potential role in diagnosis and monitoring plaque in AS progression. Our discovery of m6A DNA in mammalian cells sheds light on epigenetic modification during human CVD.

## Methods

### Ethics statement

This randomized hospital-based and case–control study was performed between October 2018 and June 2019. The protocol conformed to the ethical guidelines of the 1975 Declaration of Helsinki and was approved by the Ethics Committee of the Second Affiliated Hospital of Wenzhou Medical University in October 2018 (No. L-2018-43). Signed informed consent was obtained from each participant before entering this study.

### Study population

In this case–control study, we randomly and blindingly enrolled 207 patients with AS (116 men; mean age 60.00 ± 12.70 years) from the Second Affiliated Hospital of Wenzhou Medical University. Using creative research-systems survey software, we randomly selected 141 sex- and age-matched healthy individuals who were visiting the hospital for a health examination as the normal control group.

AS was defined by the presence at least one atherosclerotic lesion or infarct in the aorta artery, carotid artery, cerebrovascular region, coronary artery or kidney artery, confirmed by CT arteriography, aortic angiography, left ventriculography or MRI. We also defined significant clinical signs and symptoms as AS: chest pain or pressure, sudden numbness or weakness in arms or legs, difficulty speaking or slurred speech, temporary loss of vision in one eye, drooping muscles in the face, leg pain when walking (claudication), high blood pressure, kidney failure, or stroke. We excluded patients with current infections, genetic diabetes, uremia, active malignancies, aneurysm, inflammatory diseases, or iatrogenic and traumatic aortic dissections. We also excluded pregnant women and patients with myocardial infarction, stroke, coronary revascularization, or peripheral vascular surgery during the preceding 6 months.

### Laboratory analyses

A total of 4 ml fasting cubital venous blood was drawn from each participant and used to separate serum. Leukocyte cell samples were separated into new tubes for DNA analysis. Serum concentrations of triglyceride (TG), total cholesterol (TC), Hcy, blood glucose, high-density lipoprotein cholesterol (HDL), low-density lipoprotein cholesterol (LDL), hemoglobin A1 (HbA1), fasting blood glucose, uric acid, blood urea nitrogen (BUN), g-glutamyl transpeptida, apolipoprotein B (ApoB), creatinine, BUN, glucose, high-sensitivity C-reactive protein, folate, vitamin B12 (VB12), and creatinine (Cr) were measured by routine techniques using an automated analyzer (Beckman AU5800) at the Clinical Laboratory Department of the Second Affiliated Hospital of Wenzhou Medical University. The intra- and inter-assay coefficients of variation were <10% for all biochemical variables.

### Quantification of DNA methylation

Genomic DNA was extracted from peripheral blood specimens, aortic root and cell lines by using the DNeasy Blood & Tissue Kit (Qiagen, CA). The integration of genomic DNA was confirmed on an agarose gel and measured by using a NanoDrop spectrophotometer. DNA methylation of 5mC and m6A was assessed by using the MethylFlash m6A DNA Methylation ELISA Kit (Colorimetric) and MethylFlash Global DNA Methylation (5mC) ELISA Easy Kit (Colorimetric) following the manufacturer’s instructions (Epigentek, NY). Briefly, the methylated fraction of 100 ng of leukocyte genomic DNA was recognized by the 5mC or m6A antibody and colorimetrically quantified by an ELISA-like reaction. The percentage of methylated DNA was calculated as a proportion of optical density. Relative quantification was used to calculate the percentage of 5mC or m6A in total leukocyte DNA following the manufacturer’s instructions. Methylated (positive) and unmethylated (negative) control DNA was incubated in strip wells with a specially developed solution to promote DNA binding and adherence to the sample well. Each sample was run in duplicate.

### CIMT measurements

Both left and right carotid arteries were measured according to a standardized protocol by one blinded trained sonographer who used a B-mode ultrasonic diagnostic apparatus (GE, USA). Bilateral carotid arteries were scanned with participants in the supine position with the neck hyperextended. Technical data including CIMT were obtained. The CIMT was determined as the distance from the media-adventitia interface to the intima-lumen interface on the far wall in a region free of plaques. CIMT 1.0–1.3 mm was defined as carotid AS; >1.3 mm or 0.5 mm thicker than the adjacent site or >1.5 times of that of adjacent site was considered presence of carotid plaques. Each measurement was repeated three times. The intra- and inter-measure coefficient of variation was <3%.

### Primary cells and cell lines and treatment

Primary human umbilical vein endothelial cells (HUVECs) were isolated from human umbilical cord within 10 h of delivery as we previously described^28,29^. The cells were maintained and subcultured in Medium 199 (Sigma-Aldrich, USA) containing 10% fetal bovine serum (FBS, Hyclone, USA), 20 mM 4-(2-hydroxyethyl)-1-piperazineethanesulfonic acid (Hepes), 10 μg/ml endothelial cell growth supplement, 15 IU/ml heparin (Baxter, Austria), and 100 μM penicillin–streptomycin (PS; Sigma, Austria). HUVECs between passages 4 and 7 were used for all experiments. The human monocytic cell line, THP1 cells, was obtained from American Type Culture Collection (TIB-202). According to the manufacturer’s instructions, THP1 cells were cultured in RPMI-1640 supplemented with 10% FBS, 20 mM Hepes, and 100 μM PS. All cell cultures were maintained in a humidified 5% CO_2_ incubator at 37 °C. Both of cells were authenticated by STR profiling and tested for mycoplasma contamination before used.

HUVECs and THP1 cells at 80% confluence and containing 2% FBS were used for treatment or transfection. Cells were treated with concentrations of ox-LDL (0, 5, 10, 25, 50, and 100 mg/L) (Athens Research & Technology, USA) for 24 h. Cells with siRNA for ALKBH1 and HIF1α or control (siRNA-A) (Santa Cruz Biotechnology, USA) were transfected with Lipofectamine RNAiMAX Reagent (Invitrogen, CA) as we described^27^.

### Animal model, tissue sample preparations, and immunofluorescence analysis

Eight-week-old male apolipoprotein E-knockout (ApoE^−/−^) C57BL/6J mice (purchased from Nanjing Biomedical Research Institute of Nanjing University, China) were randomly and blindingly fed a ND or WD (*n* = 19/group) for 6 months. Mice were anesthetized by intraperitoneal injection of pentobarbital (50 mg/kg body weight). The degree of anesthesia was monitored with a foot reflex. Body temperature was maintained at 37 °C with a heating pad. About 1 ml blood was drawn and separated into serum and leukocytes. Subsequently, aortic root tissues were isolated from four mice from each group for reverse transcription polymerase chain reaction (RT-PCR). A total of 15 mice in each group were perfused with 10 ml phosphate buffered saline for washing blood and with 4% paraformaldehyde (PFA) for prefixing by using heart puncture (left ventricle). The whole aortas including aortic root, thoracic aorta and abdominal aorta were isolated and further fixed in 4% PFA at 4 °C for 2 h. Then, the whole aortas were embedded in optimal cutting temperature compound with orientation for front sections. Frozen aortic root sections (5 μm) were collected on glass slides for immunofluorescence (IF) co-staining and Oil-red O staining as described^27^. The largest atherosclerotic lesion area was quantified by Oil-red O staining. For IF co-staining, tissue sections were incubated with two primary antibodies for N6AMT1 (Proteintech: 16211-1-AP), ALKBH1 (Abcam: ab195376) or 5-methylcytosine (5mC, Abcam: ab10805) and the biomarker of the endothelium (vWF, Proteintech: 66682-1-Ig), vascular smooth muscle cells s(α-SMA, Abcam: ab32575) or microphages (CD68, Abcam: ab955) at 4 °C overnight, then secondary antibodies conjugated with Alexa 568 or 488 fluorescence dyes for 1 h at room temperature. Representative IF images were visualized by fluorescence microscopy (Olympus, Tokyo) and relative quantification was analyzed by using Image-Pro Plus 6.0. All treatment of laboratory animals and experimental procedures were approved by the Institutional Authority for Laboratory Animals Care of the Second Affiliated Hospital of Wenzhou Medical University. All animal procedures confirmed to the guidelines from Directive 2010/63/EU of the European Parliament on the protection of animals used for scientific purposes or the NIH guidelines.

### Quantitative RT-PCR

Total RNA from peripheral blood cells, cultured cells or AS tissue was isolated by using TRIzol reagent (Invitrogen, USA) and transcribed into cDNA by using a reverse transcription kit (Promega, USA). qPCR reactions involved the SYBR Green I fluorescence kit and an Mx3000 Multiplex Quantitative PCR System (Stratagene, La Jolla, CA). We used mouse brain tissue RNA as the positive control and water as the blank control. The relative expression of N6AMT1, ALKBH1, and MIAT was normalized to their corresponding normal controls. Relative changes in expression were calculated by the 2^−∆∆^CT method (with GADPH as the internal control). All qRT-PCR analyses were performed with biological triplicates for each sample. The primer sequences used for PCR analyses based on human or mouse genes are in Table S1.

### Western blot analysis

Protein was extracted by using RIPA Buffer (Sigman, China) and quantified by using the Bicinchoninic Acid Protein Assay Kit (ThermoFisher Scientific, USA). An amount of 30 µg total protein per sample was separated by sodium dodecyl sulfate polyacrylamide gel electrophoresis and transferred to polyvinylidene fluoride membranes. The blots were incubated overnight with primary antibodies for N6AMT1 (Proteintech: 16211-1-AP), ALKBH1 (Abcam: ab195376), HIF1α (Abcam: ab1) and GAPDH (ab:8245) at 4 °C, then goat anti-rabbit or mouse IgG-HRP antibody at room temperature for 1 h. The positive bands were visualized by using Pierce ECL (ThermoFisher Scientific, USA) according to the manufacturer’s protocols.

### Luciferase reporter assay

The functional m6A binding sites were accessed from the website https://www.ncbi.nlm.nih.gov/geo/query/acc.cgi?acc=GSE118093. Potential HIF1α binding sites were identified on the human lncRNA MIAT promoter by using the Binding Site Scanner (http://nrmotif.ucr.edu) based on the Support Vector Machine (SVM). The 5′-promoter fragment of the human lncRNA MIAT locus (−1940 to +166 bp) was amplified by PCR and cloned into the pGl3-basic plasmid (Promega, USA) at KpnI and BamHI sites^30,31^. Serial deletion of MIAT constructs as well as a construct with HIF1α motif deletion were generated by using the Q5 Site-Directed Mutagenesis Kit (NEB) as per the manufacturer’s protocol. All constructs were confirmed by sequencing. Co-transfections were performed with the jetPEI-HUVEC DNA transfection reagent (Polyplus, USA) containing Renilla luciferase (Promega, E2231) bearing MIAT-pGl3-basic plasmids. Luciferase activity was assessed at 24 h after transfection by using the Dual-Glo Luciferase Assay System (Promega, E1980). The sequences of primers for serial constructs are in Table S1.

### Chromatin immunoprecipitation (ChIP)-qPCR

ChIP assay involved using the Simple Chip Enzymatic Chromatin immunoprecipitation kit (Cell Signaling Technology, USA) based on the manufacturer’s protocol. Briefly, treated HUVECs were first cross-linked with 1% formaldehyde, quenched by glycine, then digested with micrococcal nuclease. An amount of 2% lysates was used as an input reference. The lysates were incubated with 5 μg anti-m6A antibody (EMD Millipore ABE572, USA), HIF1α antibody (Santa Cruz Biotechnology, CA, USA) or goat anti-mouse IgG (ab205719, Abcam, USA) for immunoprecipitation. Then immunoprecipitates were treated with Protein G Agarose Beads overnight at 4 °C with gentle shaking. The immunoprecipitated DNA samples were cross-linked reversed, purified and amplified by PCR with their specific primers (Table S1).

### Statistical analysis

All analyses were performed with SPSS 20.0 (SPSS Inc.). Two-tailed *P* < 0.05 was considered statistically significant. Statistical significances were classified as *P < 0.05; **P < 0.01; ***P < 0.001. The data are expressed as mean ± standard deviation (SD). For sex- and age-matched case–control samples, m6A and 5mC levels were first evaluated for normality of distribution. Continuous data are estimated the variation within each group and expressed with mean ± SD. The comparison could be performed if the variance between groups are similar. Comparison of two groups involved independent-samples *t* test (unpaired). Comparison of three or more groups involved ANOVA, followed by Bonferroni’s post hoc multiple comparison tests or Dunnett’s test. Spearman correlation assay was used to assess correlation. Linear and multivariable regression analysis was used to examine the association of clinical and biological characteristics and DNA methylation with or without adjustment for age and sex. Logistic regression was used to assess the association of clinical factors with DNA methylation and risk of plaque.

## Supplementary information

Supplementary Figure Legends

Figure Suppl-1

Figure Suppl-2

Figure Suppl-3

Supplementary Table Legends

Supplement table
