# Supplementary material for: Association of N6-methyladenine DNA with plaque progression in atherosclerosis via myocardial infarction-associated transcripts
Source: Cell Death Dis. 2019 Dec 4;10(12):909. doi: 10.1038/s41419-019-2152-6 (PMC6892866; doi:10.1038/s41419-019-2152-6)
Supplement: Supplementary file 1 — Supplementary Figure Legends [file 41419_2019_2152_MOESM1_ESM.docx]

**Supplementary Figure Legends:**

**Figure S1. No significant association of leukocyte 5mC DNA levels with plaque progression in patients with clinical atherosclerosis (AS).** (A) The overall difference in leukocyte 5mC level in AS patients compared with normal controls (N). Data are mean ± SD and were compared by unpaired t-test. (B-D) Spearman correlation coefficients for leukocyte 5mC level correlated with age (B), carotid plaque size (C) and carotid intima media thickness (CIMT) (D).

**Figure S2. ALKBH1 and N6AMT1 did not differ in vascular smooth muscle cells (VSMCs) or microphages of aortic root plaque between mice treated with normal diet (ND) and western diet (WD).** (A-D) Representation immunofluorescence co-staining and quantification of ALKBH1 (A, B) and N6AMT1 (C, D) in VSMCs and microphages of frontal sections from aortic root (n=10 for ND; n=15 for WD). Scale bar: 50 µm. Alpha-smooth muscle actin (α-SMA) and CD68 were used as VSMC and microphage markers, respectively. DAPI was the nucleic marker. Data are mean ± SD and were compared by unpaired t-test.

**Figure S3. Changes in leukocyte and vascular 5mC level in mice with AS fed a WD. (A) Difference in leukocyte 5mC level between male ApoE^-/-^ mice (8 weeks old) fed a WD and ND.** (B) Spearman correlation between leukocyte 5mC level and plaque thickness of aortic root (n=15). (C-E) Representation immunofluorescence co-staining and quantification of 5mC level in endothelial cells (ECs), VSMCs and microphages of frontal sections from aortic root (n=10 for ND; n=15 for WD). Scale bar: 50 µm. von Willebrand factor (VWF), alpha-smooth muscle actin (α-SMA) and CD68 were the EC, VSMC and microphage markers, respectively. DAPI was the nucleic marker. Data are mean ± SD and were compared by unpaired t-test in A, C, D and E. Rel., Relative.
