# Supplementary figures and images for: Association of N6-methyladenine DNA with plaque progression in atherosclerosis via myocardial infarction-associated transcripts
Source: Cell Death Dis. 2019 Dec 4;10(12):909. doi: 10.1038/s41419-019-2152-6 (PMC6892866; doi:10.1038/s41419-019-2152-6)

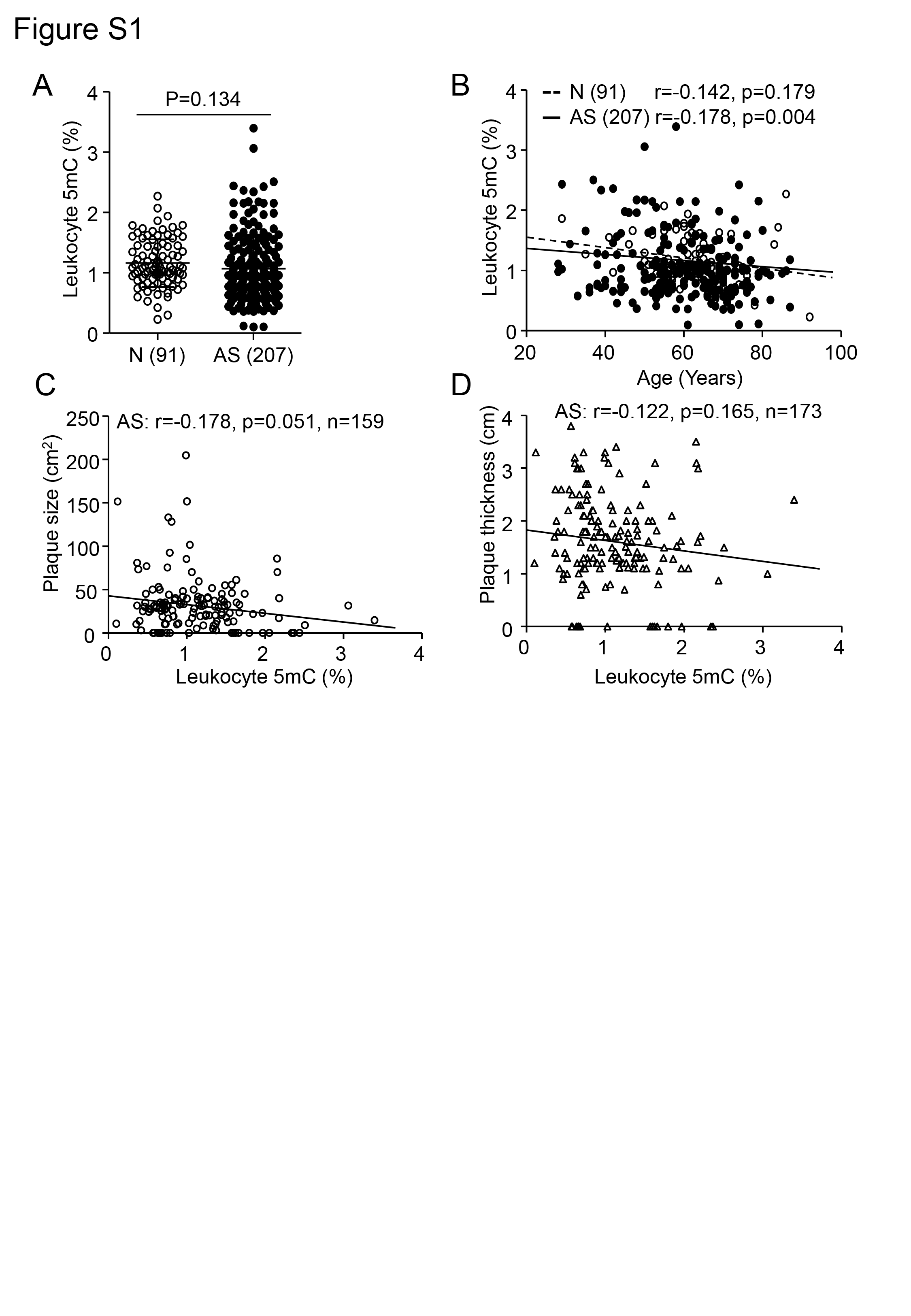

Supplement: Supplementary file 2 — Figure Suppl-1 [file 41419_2019_2152_MOESM2_ESM.tif]

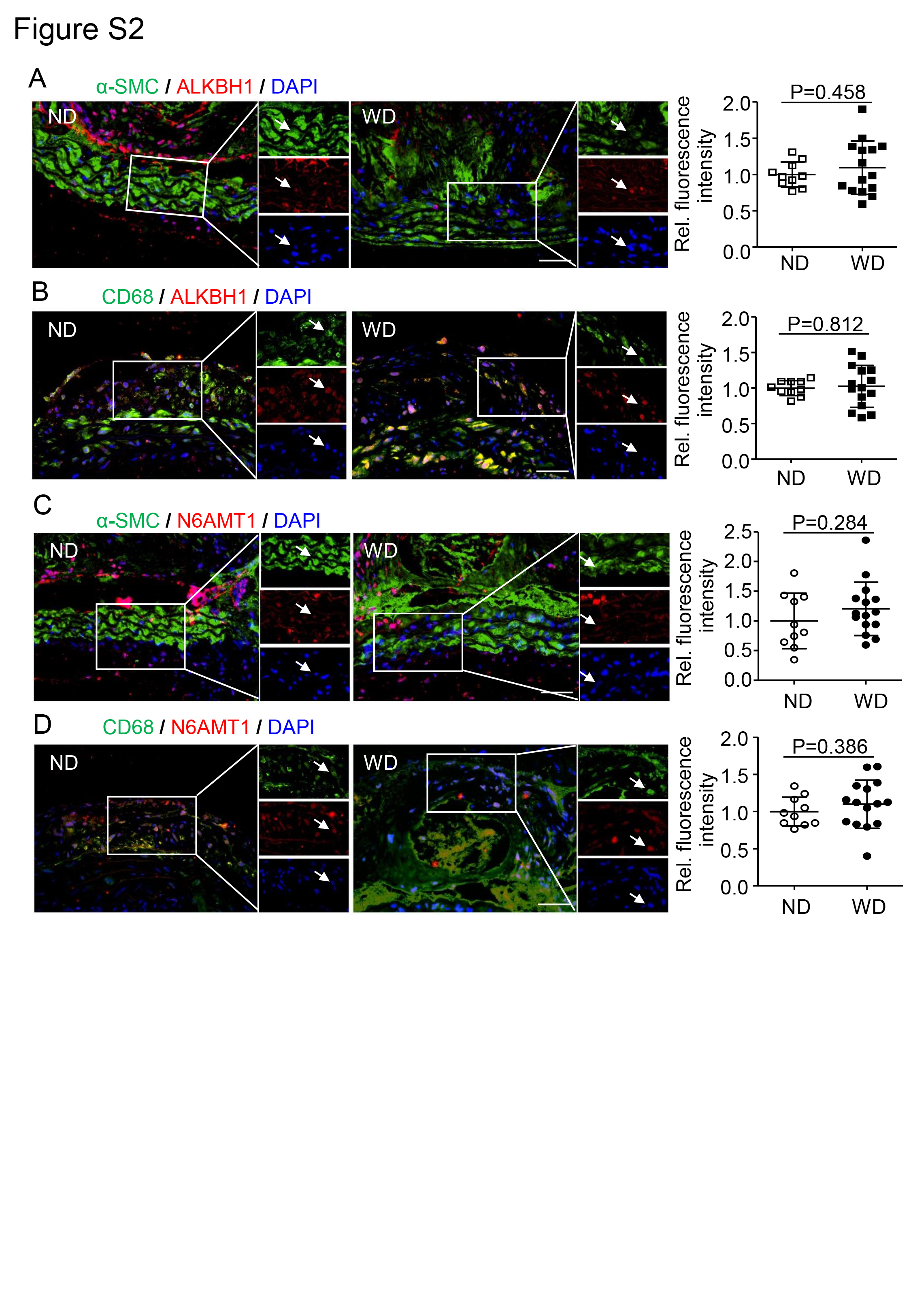

Supplement: Supplementary file 3 — Figure Suppl-2 [file 41419_2019_2152_MOESM3_ESM.tif]

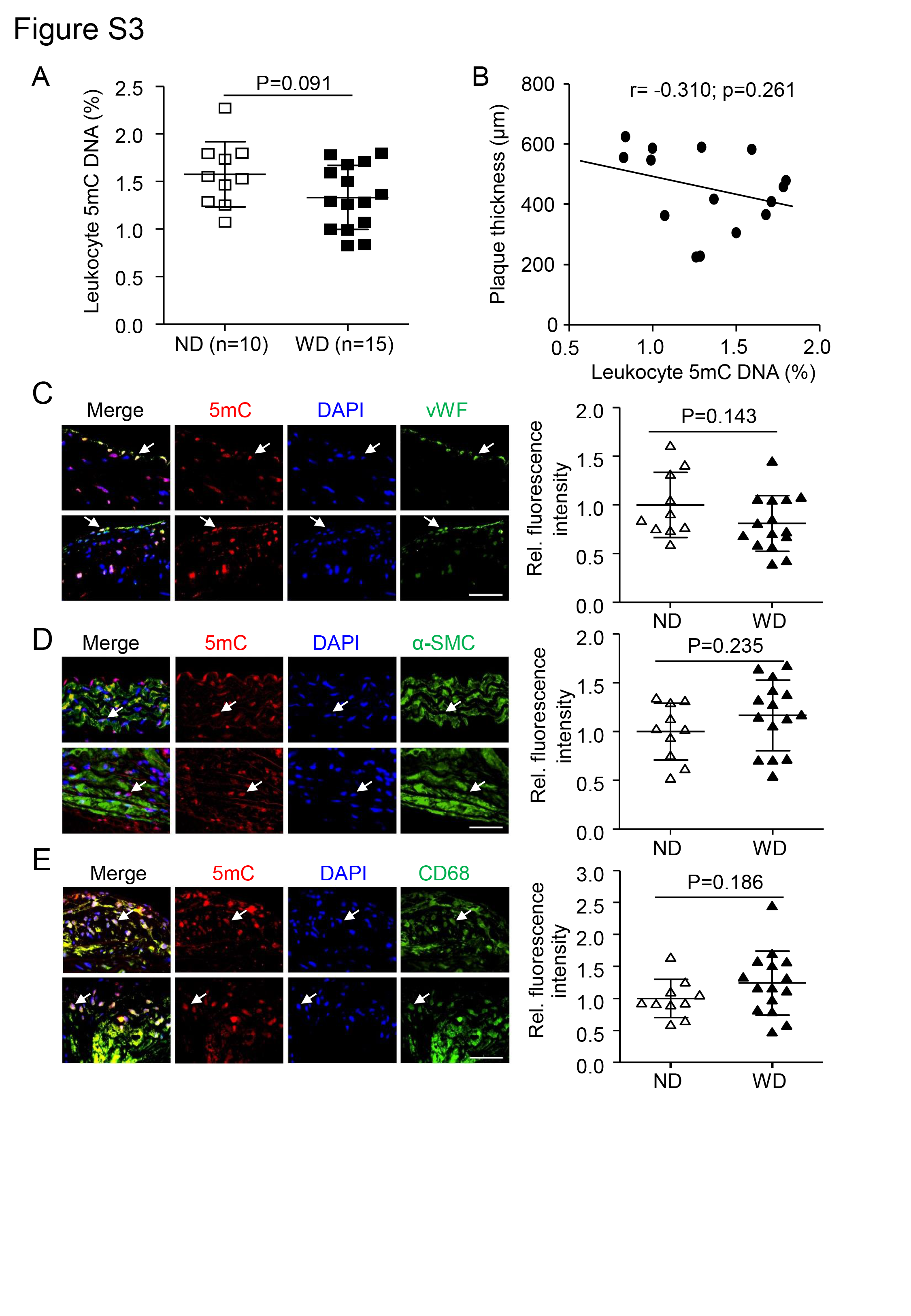

Supplement: Supplementary file 4 — Figure Suppl-3 [file 41419_2019_2152_MOESM4_ESM.tif]
