# Supplementary material for: Association of N6-methyladenine DNA with plaque progression in atherosclerosis via myocardial infarction-associated transcripts
Source: Cell Death Dis. 2019 Dec 4;10(12):909. doi: 10.1038/s41419-019-2152-6 (PMC6892866; doi:10.1038/s41419-019-2152-6)
Supplement: Supplementary file 5 — Supplementary Table Legends [file 41419_2019_2152_MOESM5_ESM.docx]

**Supplementary Table Legends:**

Table S1. Complete list of primers used in this work. Sequences are from the GenBank.
