# Supplementary material for: Association of N6-methyladenine DNA with plaque progression in atherosclerosis via myocardial infarction-associated transcripts
Source: Cell Death Dis. 2019 Dec 4;10(12):909. doi: 10.1038/s41419-019-2152-6 (PMC6892866; doi:10.1038/s41419-019-2152-6)
Supplement: Supplementary file 6 — Supplement table [file 41419_2019_2152_MOESM6_ESM.docx]

Table S1.

| **Assay Name** | **Reverse primer sequence (5’–3’)** | **Products** |
| --- | --- | --- |
| **RT–PCR** | | |
| Human MIAT | Forward: GGACGTTCACAACCACACTG | 128bp |
|  | Reverse: TCCCACTTTGGCATTCTAGG |  |
| hALKBH1 | Forward: AGAAGCGACTAAACGGAGACC | 117bp |
|  | Forward: GGGAAAGGTGTGTAATGATCTGC |  |
| hN6AMT1 | Forward: GCAGGGGAGAACTTCGCTAC | 106bp |
|  | Forward: CAGCGCGTTCAAAAGCAGAAA |  |
| Human GAPDH | Forward: GGATTTGGTCGTATTGGG | 205bp |
|  | Reverse: GGAAGATGGTGATGGGATT |  |
| Mouse Miat | Forward: ACAACACCAACCCACAAGGT | 179bp |
|  | Reverse: CAGGACTGTTGTGCCAAAGC |  |
| mN6AMT1 | Forward: AGCCGCATGTACCTTGGAAA | 154bp |
|  | Forward: TACCTCTTCAGGCGGAGTCA |  |
| mALKBH1 | Forward: AAGCGAAGACCCCGAAGTTTA | 145bp |
|  | Forward: CAGTGGCGACTTGCTCTGA |  |
| Mouse Gapdh | Forward: CGACTTCAACAGCAACTCCCACTCTTCC | 285bp |
|  | Reverse: TGGGTGGTCCAGGGTTTCTTACTCCTT |  |
| **MIAT luciferase report assay** | | |
| -1940bp~+166bp | Forward: GGGGTACCCCGGTGTGGAGCTTGCCTAAGGT | 2106bp |
|  | Reverse: GTTCCCCGTTAGGGGACAAGCGGGATCCCG |  |
| -1389bp~+166bp | Forward: GGGGTACCCCGCACCACGCTTGGCTAATTTT | 1555bp |
|  | Reverse: GTTCCCCGTTAGGGGACAAGCGGGATCCCG |  |
| -661bp~+166bp | Forward: GGGGTACCCCGAGAGTGAGGTCCCATCTCTA | 827bp |
|  | Reverse: GTTCCCCGTTAGGGGACAAGCGGGATCCCG |  |
| -95bp~+166bp | Forward: GGGGTACCCCGCATTAAAATTTCATGGGCGC | 261bp |
|  | Reverse: GTTCCCCGTTAGGGGACAAGCGGGATCCCG |  |
| HIF1α del | Forward: AATTGGATTTCTCAAGCAATGTGGATGC | 2096bp |
|  | Reverse: CCACATTGCTTGAGAAATCCAATTCGAG |  |
| **MIAT ChIP assay** | | |
| m6A ChIP-1 | Forward: TTTTGCTCTGTTGCCCATGC | 130bp |
|  | Reverse: ATGGTAGTGTGTGCCTGTGG |  |
| m6A ChIP-2 | Forward: GCCTGGCCTCGAATTGGATT | 93bp |
|  | Reverse: GGGATGCTAGGAAGTGGTCC |  |
| m6A ChIP-3 | Forward: TGCACTGCTCCTGGATTCTG | 113bp |
|  | Reverse: CTGCTCTCTTGCAAACGCTG |  |
